# Nonclassical Congenital Adrenal Hyperplasia and Pregnancy

**DOI:** 10.1155/2015/296924

**Published:** 2015-10-08

**Authors:** Neslihan Cuhaci, Cevdet Aydın, Ahmet Yesilyurt, Ferda Alpaslan Pınarlı, Reyhan Ersoy, Bekir Cakir

**Affiliations:** ^1^Department of Endocrinology and Metabolism, Faculty of Medicine, Yildirim Beyazit University, 06800 Ankara, Turkey; ^2^Department of Genetics, Dıskapı Yildirim Beyazit Education and Research Hospital, Ankara, Turkey

## Abstract

*Objective*. The most common form of congenital adrenal hyperplasia (CAH) is 21-hydroxylase (21-OH) deficiency due to mutation of the *CYP21A2* gene. Patients with nonclassical CAH (NC-CAH) are usually asymptomatic at birth and typically present in late childhood, adolescence, or adulthood with symptoms of excessive androgen secretion. Subfertility is relative in NC-CAH, but the incidence of spontaneous miscarriage is higher. Here, we report a previously undiagnosed female who gave birth to a normal male child and is planning to become pregnant again. *Case Report*. A 32-year-old female was referred to our clinic for obesity. Her medical history revealed that she had had three pregnancies. She was planning to become pregnant again. Her laboratory results revealed that she had NC-CAH. Since her husband is the son of her aunt and she had miscarriages and intrauterin exitus in her history, their genetic analyses were performed. *Conclusion*. Since most patients with NC-CAH have a severe mutation, these patients may give birth to a child with the classical CAH (C-CAH) if their partner is also carrying a severe mutation. Females with NC-CAH who desire pregnancy must be aware of the risk of having an infant with C-CAH.

## 1. Introduction

Congenital adrenal hyperplasia (CAH) is a group of autosomal recessive disorders that are characterized by impaired cortisol synthesis and adrenal androgen excess, caused by a deficiency in one of the enzymes necessary for cortisol production [1–3]. The most common form of CAH is 21-hydroxylase (21-OH) deficiency due to mutation of the* CYP21A2* gene, which encodes the adrenal steroid 21-OH enzyme and is located on chromosome 6p21.3 [1, 4–9].

It can be defined as classical CAH (C-CAH), or nonclassical or late-onset CAH (NC-CAH). C-CAH can be of either the salt wasting (SW) or simple virilizing (SV) type.

NC-CAH 21-OH deficiency is much more common than C-CAH [8], with a reported prevalence of 0.1–0.4% in the general population [10]. It is also more frequent in certain ethnicities such as Ashkenazi Jewish, Mediterranean, Middle-Eastern, and Indian populations [11].

Patients with NC-CAH are usually asymptomatic at birth [7] and typically present in late childhood, adolescence, or adulthood with symptoms of excessive androgen secretion [12]. In adolescent and adult females, the symptoms of hyperandrogenism include hirsutism, acne, menstrual irregularity, androgenic alopecia, and impaired fertility [2, 6].

Subfertility is relative in NC-CAH, but the incidence of spontaneous miscarriage is higher [2]. However, few data regarding the fertility of NC-CAH patients are available [13].

Many females with NC-CAH conceive spontaneously, whereas others have ovulatory infertility but respond to glucocorticoid (GCC) or GCC plus clomiphene citrate treatment. However, the risk of spontaneous miscarriage is higher in these females compared with normal females (>25 versus 10–15%, resp.) [13–18]. Approximately 70% of individuals with NC-CAH have a point mutation of Val281Leu at exon 7, which prevents 20–50% of enzyme activity [19]. Previous studies reported that 27–76% of patients with NC-CAH have a severe mutation [13]. If their partner also carries a severe mutation, these patients might conceive a child with C-CAH [13].

In this study, we report a previously undiagnosed female who gave birth to a normal male child and is planning to become pregnant again.

## 2. Case

A 32-year-old female was referred to our clinic for obesity. She had hypothyroidism and was using L-thyroxin replacement therapy. Her medical history revealed that she had had three pregnancies: one had resulted in a healthy boy, one had resulted in ectopic pregnancy with twins and intrauterine exitus, and the latest, which had occurred 4 months earlier, had been terminated by miscarriage. She was planning to become pregnant again.

A physical examination revealed that her body mass index (BMI) was 26 kg/m^2^. She had no purple striae or a buffalo hump and no hirsutism: her Ferriman-Gallwey score was 5. Laboratory results related to obesity revealed normal thyroid function tests, insulin resistance calculated using the HOMA-index was 1.8, and her initial random serum cortisol level was 17.8 *μ*g/dL. Because of the high cortisol level, 1 mg dexamethasone suppression test (DMST) was applied, which successfully suppressed her cortisol level to 0.6 *μ*g/dL. A follicular phase hormonal evaluation identified elevated 17-OHP (12.8 ng/mL) and ACTH (56 pg/mL) levels. The other hormonal profiles were all normal, including follicle-stimulating hormone (FSH), luteinizing hormone (LH), estradiol (E2), progesterone, prolactin (PRL), dehydroepiandrosterone sulfate (DHEA-SO4), and testosterone. There was no pathology on pelvic ultrasonography. We next performed an ACTH stimulation test and found 17-OHP levels at basal, 30 and 60 minutes of 14, 21, and 26 ng/mL, respectively, which confirmed the diagnosis of NC-CAH.

During these processes, we learned that she was a carrier of the* MTHFRC677* mutation. Before admission to our hospital, karyotype analysis was performed due to the previous miscarriage and intrauterine exitus. The results revealed a normal constitutional karyotype of 46, XX. Her husband, who is the son of her aunt (in other words their mothers are sisters), also had a normal constitutional karyotype of 46, XY. Because we confirmed the diagnosis of CAH and she is related to her husband, we also studied his laboratory tests, but no pathology was found. Genetic analyses were then performed on both the patient and her husband. In the genetic test results the patient had revealed a compound heterogeneous mutation of Q318X and P453S in the* CYP21A2* gene, and her husband revealed a 453S heterogeneous mutation in the* CYP21A2* gene. They have the same P453S mutations in the* CYP21A2* gene. Because both parents were carriers of* CYP21A2* mutations, we recommended preconception genetic counseling to the couple. They were informed of the risks to the fetus and newborn child, and fetal sampling was recommended if she became pregnant. Many patients with NC-CAH are asymptomatic and current recommendations argue against treatment for those without symptoms [19]. Treatment with glucocorticoid therapy is typically only recommended for those individuals with symptomatic hyperandrogenism [19]. Nonpregnant adults may be treated with the longer-acting DM or prednisone, alone or in combination with hydrocortisone [8]. Hydrocortisone is the preferred treatment of pregnant women affected with CAH, because unlike DM, it is metabolized by the placental enzyme 11-*β* OH steroid dehydrogenase II and does not affect the fetus [8]. We did not start treatment before pregnancy, as suggested by the Endocrine Society guidelines [3], but, instead, planned to begin treatment when pregnancy occurred.

## 3. Discussion

Unlike the situation with C-CAH, few studies have assessed fertility in females with NC-CAH [13]. Bidet et al. analyzed 190 females with NC-CAH, 95 of whom wanted to become pregnant [13]. They reported that 187 pregnancies occurred in 85 females, which resulted in 141 births from 82 individuals. A total of 99 pregnancies (52.9%) occurred before diagnosis with NC-CAH (96 spontaneous and three with ovulation inducers), and 88 occurred after diagnosis (11 spontaneously and 77 with hydrocortisone treatment). The miscarriage rate was 6.5% and 26.3% in the patients treated with GCC and untreated patients, respectively. The authors also reported that 1.5% of the infants were born with C-CAH [13]. Birnbaum and Rose [20] observed 12 pregnancies among 22 NC-CAH patients who desired pregnancy, whereas Feldman et al. [16] observed that, of 20 patients wanting pregnancy, 10 had conceived before diagnosis, and nine after NC-CAH diagnosis and hydrocortisone treatment. Some studies have also assessed fertility in males with NC-CAH [19]. Although oligospermia has been reported, gonadal functions and sperm counts are relatively normal in males with NC-CAH, which suggests that NC-CAH might be underdiagnosed [21, 22]. One study reported that affected males are usually asymptomatic, and they are commonly diagnosed after the diagnosis of a female family member [23], as seen in our patient's husband.

Females with NC-CAH who desire pregnancy must be aware of the risk of having an infant with C-CAH. The incidence of C-CAH is 1 : 10,000–1 : 20,000, and the incidence of carriers in the general population is 1 : 50–1 : 71 (median 1 : 60) [19]. As such, the chance of having a child with C-CAH in a patient with C-CAH is 1 : 120 [19, 24]. Since most patients with NC-CAH carry a severe* CYP21A2* mutation in one allele, they are at risk of having a child with C-CAH [19]. Therefore, parents with NC-CAH would be predicted to have a 1 : 240 chance of having a child with C-CAH (1/60 × 1/2) × (1 × 1/2) [3, 19, 24]. Furthermore, because two-thirds of patients with NC-CAH are compound heterozygotes, the predicted incidence is ~1 : 360 (1/60 × 1/2) × (2/3 × 1/2) [19]. One study that investigated the pregnancy outcomes of 101 females with NC-CAH determined that the risk of having a birth with C-CAH was much higher (2.5%) and that at least 15% of offspring would have NC-CAH [15]. In our case, both parents carried heterogeneous* CYP21A2* mutations: the female had Q318X and P453S mutations, whereas her husband carried a 453S mutation. Seventy percent of NC-CAH individuals carry a point mutation, Val281Leu, at exon 7 [19]. P453S and R339H are also associated with NC-CAH; also R369W and I230T are two novel mutations associated with NC-CAH [19]. Additionally, Q318X mutation has been reported in association with CAH [6]. Large deletions and a splicing mutation that ablate enzyme activity comprise about 50% of C-CAH alleles [3]. A nonconservative amino substitution in exon 4 is associated with simple virilizing C-CAH [3]. Because they have the chance of giving birth to a child with C-CAH, our parents were offered preconception genetic counseling.

Prenatal treatment for CAH is still experimental. The aims of treatment include the genital virilization of the fetus, reducing the anxiety of the parents who might have a child with ambiguous genitalia [24]. However, prenatal treatment does not prevent the need for lifelong GCC and mineralocorticoid (MCC) replacement therapy, and intensive medical monitoring during infancy and later life, or the potential life-threatening salt-wasting crises that occur when postnatal treatment is discontinued [24]. Dexamethasone (DM) is commonly used because it binds minimally to cortisol-binding globulin (CBG) in the maternal blood and, unlike hydrocortisol, it is not inactivated by placental 11-beta hydroxysteroid dehydrogenase type II [6, 8]. Consequently, it crosses the placenta and suppresses ACTH secretion [8]. If karyotype or DNA analyses reveal that the fetus is male or an unaffected female, respectively, treatment is discontinued [8]. Because fetal genital virilization begins 6-7 weeks after conception, treatment must be started as soon as the female learns she is pregnant [3, 24]. However, because chorionic villous biopsies can be obtained after 10–12 weeks for genetic diagnosis and the procedure takes additional time, all pregnancies deemed to be at risk for virilizing CAH are treated, even though only 1 in 4 is affected, and only 1 in 8 affected fetuses is female [3, 6, 24]. Therefore, it was suggested that DM exposure is undesirable and unethical in 7 out of 8 fetuses (males and unaffected females) [24]; this remains controversial [24]. Because of the methodological limitations and small sample sizes, evidence for the maternal and fetal sequelae of prenatal DM treatment to accurately assess the fetuses at risk for CAH is limited or is of poor quality [3, 25]. Endocrine Society guidelines suggest that prenatal treatment should be pursued using protocols approved by Institutional Review Boards at all centers that are capable of collecting outcome data from a sufficiently large number of patients, which will allow the risks and benefits of this treatment to be defined more precisely [3]. Therefore, we did not start treatment before pregnancy occurred, as suggested by Endocrine Society guidelines [3]. In the study of New and colleagues [26], they found that the average Prader score of the fetuses treated with DM was 1.7, which was much lower than the average Prader score of 3.73 in those not treated. Although their data demonstrated no significant abnormality in the long-term medical and cognitive outcomes in the patients treated with DM prenatally, the procedures such as chorionic villus sampling and amniocentesis were invasive and also all the fetuses treated unnecessarily before the sex and the affection status of the fetus is known. In a recent study of New et al. [27], they developed a noninvasive method for early prenatal diagnosis of fetuses at risk for CAH and found that cell-free fetal DNA obtained from maternal plasma could potentially provide the diagnosis of CAH, noninvasively, so, only the affected female fetuses will be treated before the 9th week of gestation. Their study seems promising. But we decide to treat the patient when pregnancy occurs, obtain chorionic villous sampling 10–12 weeks into the pregnancy, and then manage the treatment appropriately.
